# Prevalence of Current and Past SARS-CoV-2 Infections among Police Employees in Poland, June–July 2020

**DOI:** 10.3390/jcm9103245

**Published:** 2020-10-11

**Authors:** Mariusz Gujski, Mateusz Jankowski, Jarosław Pinkas, Waldemar Wierzba, Piotr Samel-Kowalik, Artur Zaczyński, Piotr Jędrusik, Igor Pańkowski, Grzegorz Juszczyk, Kamil Rakocy, Filip Raciborski

**Affiliations:** 1 Department of Prevention of Environmental Hazards and Allergology, Medical University of Warsaw, 02-091 Warsaw, Poland; mariusz.gujski@wum.edu.pl (M.G.); piotr.samel@wum.edu.pl (P.S.-K.); filip.raciborski@wum.edu.pl (F.R.); 2 School of Public Health, Centre of Postgraduate Medical Education, 01-826 Warsaw, Poland; jpinkas@cmkp.edu.pl; 3 Central Clinical Hospital of the Ministry of the Interior and Administration in Warsaw, 02-507 Warsaw, Poland; waldemar.wierzba@cskmswia.pl (W.W.); artur.zaczynski@cskmswia.pl (A.Z.); igor.pankowski@cskmswia.pl (I.P.); 4 UHE Satellite Campus in Warsaw, University of Humanities and Economics in Łódź, 01-513 Warsaw, Poland; 5 Department of Internal Disease, Hypertension and Vascular Diseases, Medical University of Warsaw, 02-097 Warsaw, Poland; pjedrusik@wum.edu.pl; 6 Department of Public Health, Medical University of Warsaw, 02-097 Warsaw, Poland; grzegorz.juszczyk@wum.edu.pl; 7 KR Consulting, 00-001 Warsaw, Poland; rakkam@gmail.com

**Keywords:** COVID-19, SARS-CoV-2, epidemiology, serology, RT-PCR, police

## Abstract

Background: Coronavirus disease 2019 (COVID-19) is caused by severe acute respiratory syndrome coronavirus 2 (SARS-CoV-2). We aimed to determine the prevalence of current and past SARS-CoV-2 infections among police employees. Methods: This cross-sectional survey was undertaken among 5082 police employees from Mazowieckie Province, Poland. RT-PCR testing for current SARS-CoV-2 infection and serological tests (ELISA) for the presence of anti-SARS-CoV-2 IgM+IgA and IgG antibodies were performed. Results: All RT-PCR tests were negative. The anti-SARS-CoV-2 IgM+IgA index was positive (>8) in 8.9% of participants, including 11.2% women and 7.7% men (*p* < 0.001). Equivocal IgM+IgA index (6–8) was found in 9.8% of participants, including 11.9% women and 8.7% men (*p* < 0.001). The IgG index was positive (>6) in 4.3% and equivocal (4–6) in 13.2% of participants. A higher odds of positive IgM+IgA index was found in women vs. men (OR: 1.742) and police officers vs. civilian employees (OR: 1.411). Participants aged ≥60 years had a higher odds of positive IgG index vs. those aged 20–29 years (OR: 3.309). Daily vaping also increased the odds of positive IgG index (OR: 2.058). Conclusions: The majority of Polish police employees are seronegative for SARS-CoV-2 infection. Vaping and older age (≥60 years) were associated with a higher risk of SARS-CoV-2 infection.

## 1. Introduction

Coronavirus disease 2019 (COVID-19) is caused by a novel strain of coronavirus, severe acute respiratory syndrome coronavirus 2 (SARS-CoV-2), which appeared in China in 2019 [1] and evolved into the current pandemic. Although the definite laboratory diagnosis of SARS-CoV-2 infection is currently based on real-time reverse transcriptase-polymerase chain reaction (RT-PCR) testing, and RT-PCR is recommended for clinical testing in cases of suspected COVID-19 disease [2], asymptomatic infected individuals (infection carriers) who do not come to medical attention may play an important role in transmitting infection within the population [3]. As the time window for a positive RT-PCR result is short, serological testing, which provides information about whether a person has been exposed to SARS-CoV-2, may be useful for epidemiological purposes to detect the overall burden of previous infection in a given community.

Currently, two types of serological assays are available for SARS-CoV-2 testing [2]. Laboratory-based immunoassays, including enzyme-linked immunosorbent assays (ELISAs), chemiluminescentmicroparticle immunoassays, and immunometric assays, detect various classes of immunoglobulins (Ig) against SARS-CoV-2, including IgM, IgA, and IgG, can be qualitative or quantitative, and are generally performed using serum samples. Rapid diagnostic tests are typically lateral-flow assays that can be used for point-of-care testing to detect anti-SARS-CoV-2 IgG, IgM, or viral antigens and are usually performed in fingerstick blood samples, although some may use saliva or other specimen types.

The rationale for screening using tests detecting SARS-CoV-2-specific antibodies is that these antibodies develop regardless of symptoms and are present for several months after infection [4]. In addition, population data from Spain and Iceland indicate that a substantial proportion of infected persons, both asymptomatic and symptomatic, are never tested with RT-PCR in the acute phase [3,4]. The data obtained during the current SARS-CoV-2 epidemic in Poland indicate that more than 90% of infections within the workforce outbreaks, e.g., among miners, which involve younger and generally more healthy persons, were asymptomatic or oligosymptomatic [5].

Poland is a country where the coronavirus epidemic arrived relatively late. The first laboratory-confirmed COVID-19 case was reported on 4 March 2020 [6]. As of 21 September 78,330 laboratory-confirmed COVID-19 cases and 2282 related deaths were reported in Poland [7]. The settings of SARS-CoV-2 transmission during the first 2 months of the epidemic were mostly hospitals and long-term care facilities, followed by outbreaks in workplaces, including coal mines, furniture factories, and meat-processing plants [8].

Polish police officers are at an increased risk of acquiring SARS-CoV-2 infection due to their duties, including protection of public gatherings and daily verification (by direct visual contact) of compliance with mandatory quarantine rules. Nationwide, the number of quarantined persons was >160,000 at the peak in early April 2020 [9] and currently (early September 2020) is about 70,000 [10].

The aim of this study was to determine the prevalence of current and past SARS-CoV-2 infections among police employees, a high-risk population due to their professional duties, during the COVID-19 epidemic.

## 2. Materials and Methods

### 2.1. Study Design and Participants

This cross-sectional SARS-CoV-2 screening survey was carried out from 22 June to 8 July 2020 among police employees (police officers and civilian employees) from Mazowieckie Province in Poland.

Random-cluster sampling was performed. Out of 327 police units (including headquarters/police stations and departments), 170 were randomly selected. The smallest units (<5 people) were excluded from the sampling procedure. The cluster and stratified selection method was applied for sampling procedures to improve accuracy. All police employees from the randomly selected units were invited to participate in the study (8789 individuals from 170 units). Due to refusal to participate and exclusion of the smallest units, the study was finally carried out in 122 police units. The questionnaire was completed by 5363 police employees, and biological samples (swab and blood) were effectively collected from 5082 of them. The exclusion criteria include refusal to participate, lack of a signed informed consent, hospitalization, quarantine, leave, and secondment to work in another police unit that was not selected for the survey.

Participants were invited via email or the police’s internal communication system. After completing the questionnaire, each respondent received an individual ID code for personal data protection. Test samples were collected on the premises of the police units (in a dedicated room) on a day designated by the research team. Samples were collected by a nurse or a paramedic equipped with personal protective equipment according to the applicable safety procedures.

### 2.2. Measurements

#### 2.2.1. RT-PCR Testing for Current SARS-CoV-2 Infection

Nasopharyngeal swab samples were collected by a nurse or a paramedic using transport sets specially designed for collecting clinical material for the diagnosis of SARS-CoV-2 infection. The DiaPlexQ™ Novel Coronavirus (2019-nCoV) Detection Kit (SolGent Co., Ltd.; Daejeon, Korea) was used for the detection of SARS-CoV-2 RNA by RT-PCR. Virus identification (positive result) was based on the ORF1ab and N target gene regions of SARS-CoV-2. RT-PCR testing was carried out in the national reference laboratory (National Institute of Public Health—National Institute of Hygiene, Warsaw, Poland) by qualified clinical laboratory personnel specifically instructed and trained in RT-PCR techniques and in vitro diagnostic procedures. The testing procedure met the requirements of the WHO recommendations for COVID-19 laboratory testing [11].

#### 2.2.2. Serological Tests for the Presence of Anti-SARS-CoV-2 IgM+IgA and IgG Antibodies

Serum samples (up to 5 mL) were collected for the detection of anti-SARS-CoV-2 IgM+IgA and IgG antibodies using indirect immunoenzyme assay (ELISA). Commercially available COVID-19 ELISA IgG and IgM+IgA kits (Vircell S.L., Granada, Spain) were used, targeting SARS-CoV-2-specific antigens, spike glycoprotein (S), and nucleocapsid protein (N). Serological testing was carried out in the Diagnostic Laboratory of the Central Clinical Hospital of the Ministry of the Interior and Administration in Warsaw by qualified clinical laboratory personnel. The testing procedure was performed according to the test manufacturer’s instructions and met the Spanish Society for Infectious Diseases and Clinical Microbiology (SEIMC) recommendations. An in-house validation was carried out according to the validation protocol for users provided by the test manufacturer. According to the test manufacturer’s guidelines, the results were presented in a semiquantitativemanner andthe antibody index was calculated using the following formula:

Antibody index = (sample optical densities/cutoff serum mean optical densities) × 10.

Samples with the anti-SARS-CoV-2 IgM+IgA index below 6 were considered negative, those with the index between 6 and 8 were considered indeterminate/equivocal, and those with the index above 8 were considered positive. Samples with the anti-SARS-CoV-2 IgG index below 4 were considered negative, those with the index between 4 and 6 were considered indeterminate/equivocal, and those with the index above 6 were considered positive.

### 2.3. Study Questionnaire

The study used an original 30-item questionnaire adapted to this particular group of police employees. In preparation of the questionnaire, we analyzed the previously published COVID-19-oriented research, with special emphasis on the studies and reports published by the WHO [12]. The questionnaire was made available to the respondents via an internet platform. The Computer-Assisted Web Interview (CAWI) method was applied. Field control was enabled to avoid accidental missing data.

#### 2.3.1. Sample Size

The total number of police employees in the Mazowieckie Province was 17,400. The effective sample size of 5000 was assumed. Due to estimated nonresponse rate of 35–45% (the beginning of the holiday season and the presidential elections being held in Poland, which influenced the level of police unit involvement), a total of 8789 police employees from 170 police units were invited to take the survey. Finally, 5082 individuals took part in both questionnaire and laboratory parts of the study.

#### 2.3.2. Quantitative Variables

The questionnaire included several questions related to the personal characteristics, including age, size of the place of residence, living alone or with someone, and the presence of children in the respondent’s home. The participants were also asked about self-declared health status (very good, good, fair, or poor), presence of chronic diseases (yes/no), ever and current (past 6 months) tobacco or e-cigarette use, international travel in the past 3 months, and the nature of their work. The type of employment was categorized as a police officer (uniformed and armed force) or a civilian employee (nonuniformed, work closely with uniformed officers, e.g., administrative support). Based on the settings of official duties’ performance, the following categories were designated: office work, fieldwork, and both office work and fieldwork. Participants were also asked about the number of people with whom they had contact during the day, participation in the control of compliance with the quarantine rules, and participation in securing gatherings of more than 50 people.

Respondents were asked about the presence of 8 symptoms accompanying SARS-CoV-2 infections in the last 4 months (March–June 2020) (fever, cough, dyspnea/breathlessness, diarrhea, anorexia/lack of appetite, nausea or vomiting, loss of smell, and loss of taste). For the present analysis, only responses regarding the absence of symptoms within the given period were used.

For logistic regression analysis, all analyzed variables were recoded into a series of dummy variables (0–1). The questionnaire data were supplemented with available epidemiological data on the number of registered cases and deaths per 10,000 residents in individual poviats (administrative regions) of the Mazowieckie Province as of 8 July 2020, obtained from the State Sanitary Inspection [13]. These data were incorporated into the model as continuous variables.

### 2.4. Statistical Analysis

The data were analyzed using SPSS version 26 (IBM, Armonk, NY, USA, 2020), R version 4.0.2. (R Foundation for Statistical Computing, Vienna, Austria, 2020), and H2O version 3.30.0.7 (Apache License 2.0). The χ^2^ test was used to assess the significance of differences in cross-tables. The associations between continuous variables (IgM+IgA and IgG indexes) were measured by the Pearson’s linear correlation and the Spearman’s rank correlation. A logistic regression model was used to determine the strength of the effect of the analyzed factors on the risk of SARS-CoV-2 infection. Machine learning techniques were used to improve the fit of the model—GLM with the binominal function with ridge and lasso regularization (with cross-validation).

### 2.5. Ethics Approval

Samples were collected by qualified healthcare professionals (nurse or paramedic) in accordance with the standards set out in the ordinances of the Polish Minister of Health. Participation in the study was voluntary and free of charge. All participants gave written informed consent before participation in the study. The study protocol was reviewed and approved by the Ethics Review Board at the Medical University of Warsaw, Warsaw, Poland (approval number: KB/87/2020).

## 3. Results

Completed questionnaires were obtained from 5363 police employees (61%), and complete samples (nasopharyngeal swab and serum sample) were collected from 5082 police employees (33.5% females; response rate 57.8%). The mean age (SD) was 39.6 years (9.0) overall, 49.7 years (9.6) among women and 39.0 years (8.5) among men. Among participants, 79.2% were police officers and 20.8% were civil employees. Almost one-third (30.1%) of participants declared office-based work, 17.3% declared fieldwork, and 52.6% declared both office work and fieldwork. A quarter of the participants (25.9%) lived in rural areas, 44.8% lived in cities up to 500,000 inhabitants, and 29.3% lived in the city above 500,000 inhabitants (Warsaw).

The mean anti-SARS-CoV-2 IgM+IgA index was 4.4 ± 0.5 (range: 0.0–43.5). The mean anti-SARS-CoV-2 IgG index was 3.1 ± 0.4 (range: 0.0–44.8) (Table 1).

The anti-SARS-CoV-2 IgM+IgA and IgG indexes were linearly correlated at r = 0.209 (*p* < 0.001). Using rank correlation, the coefficient rho = 0.355 was obtained (*p* < 0.001).

Of those with negative anti-SARS-CoV-2 IgG index (<4), 7.6% had positive anti-SARS-CoV-2 IgM+IgA index (>8) and equivocal results were observed in 8.8%. Of those with positive anti-SARS-CoV-2 IgG index (>6), 18.0% had positive anti-SARS-CoV-2 IgM+IgA index (>8) and equivocal results were observed in 13.8% (Table 2). The differences were statistically significant (*p* < 0.001). Less than 1% of participants had both positive anti-SARS-CoV-2 IgM+IgA and IgG indexes.

**Figure 1:**
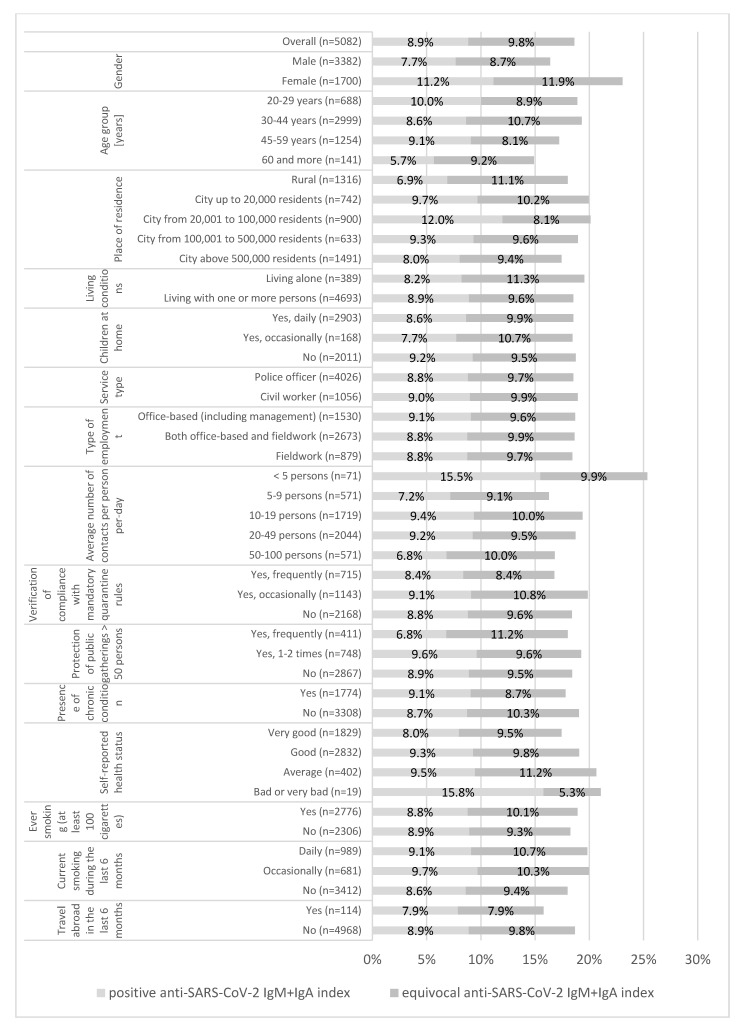
The prevalence of positive and equivocal anti-SARS-CoV-2 IgM+IgA index among 5082 police employees from Mazowieckie Province, Poland, presented by the personal and occupational characteristics.

There were no current SARS-CoV-2 infections among 5082 police employees in this study (all RT-PCR tests were negative). The anti-SARS-CoV-2 IgM+IgA index was positive (>8) in 8.9% of participants (95%CI: 8.1–9.7%) overall, in 11.2% (95%CI: 9.7–12.7%) of women, and in 7.7% (95%CI: 6.8–8.6%) of men (*p* < 0.001). An equivocal (6–8) anti-SARS-CoV-2 IgM+IgA index was found in 9.8% (95%CI: 9.0–10.6%) of participants, with a significant difference (*p* < 0.001) between women (11.9%; 95%CI: 10.4–13.5%) and men (8.7%; 95%CI: 7.8–9.7%) (Figure 1). The size of the place of residence also differentiated results in a statistically significant way (*p* < 0.01). No other variable listed in Figure 1 was significantly associated with the IgM+IgA results.

**Figure 2:**
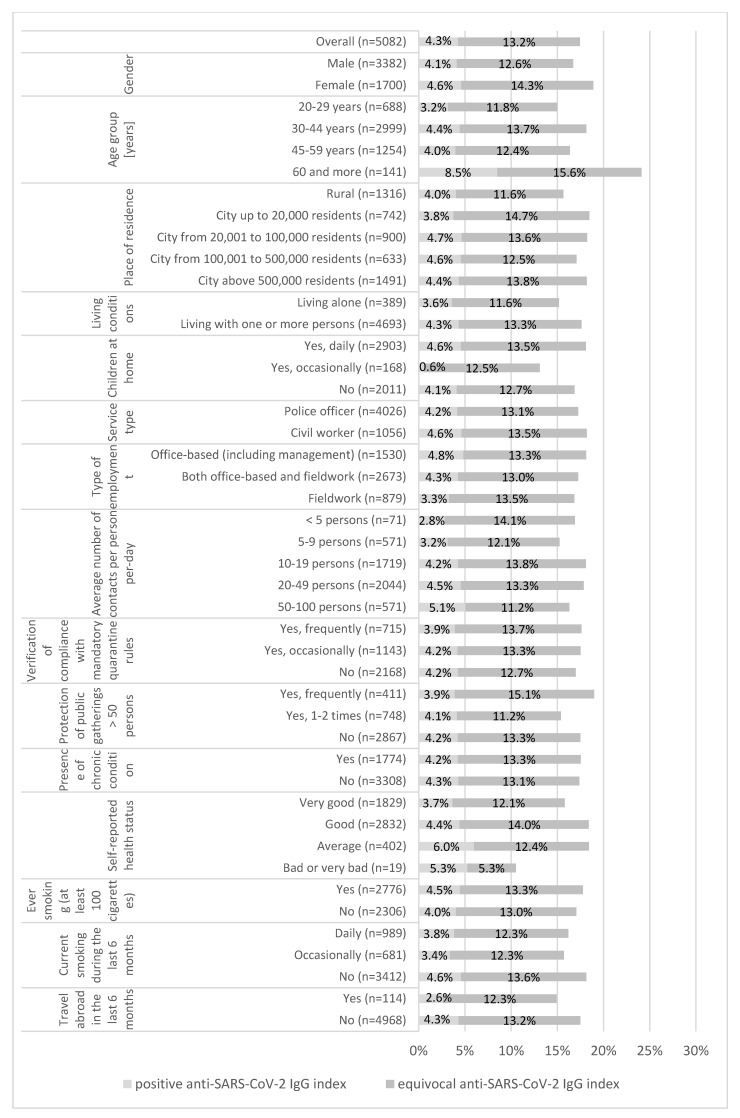
The prevalence of positive and equivocal anti-SARS-CoV-2 IgG index among 5082 police employees from Mazowieckie Province, Poland, presented by the personal and occupational characteristics.

Overall, 4.3% participants (95%CI: 3.7–4.9%) were IgG-seropositive (antibody index > 6). An equivocal (4–6) anti-SARS-CoV-2 IgG index was found in 13.2% (95%CI: 12.3–14.1%) of participants. Neither sex (*p* = 0.155) nor other variables listed in Figure 2 were significantly associated with the IgG results (Figure 2).

A logistic regression model predicting a positive anti-SARS-CoV-2 IgM+IgA index was developed (Cox and Snell R Square at 0.015 andNagelkerke R Square at 0.033). After including all variables listed in Figure 1 and Figure 2 along with the number of registered cases and deaths due to COVID-19 (per 10,000 inhabitants), only 4 variables showed a correlation with a positive anti-SARS-CoV-2 IgM+IgA index. A higher odds of a positive anti-SARS-CoV-2 IgM+IgA index was observed among women compared to men (OR: 1.742; 95%CI: 1.377–2.203), inhabitants of towns up to 20,000 residents and cities from 20,000 to 500,000 residents (OR: 1.526; 95%CI: 1.099–2.119 and OR: 1.657; 95%CI: 1.257–2.183, respectively) vs. those living in rural areas, and police officers compared to civilian employees(OR: 1.411; 95%CI: 1.004–1.981) (Table 3).

In a logistic regression model predicting a positive anti-SARS-CoV-2 IgG index (Cox & Snell R Square at 0.009, Nagelkerke R Square at 0.029), only 2 variables showed a correlation with a positive anti-SARS-CoV-2 IgG index. Compared to the age group 20–29 years, participants aged ≥60 years had higher odds of a positive anti-SARS-CoV-2 IgG index (OR: 3.309; 95%CI: 1.442–7.595). Daily vaping (e-cigarette using) also increased the odds of a positive anti-SARS-CoV-2 IgG index (OR: 2.058; 95%CI: 1.097–3.861) (Table 3).

Of the 217 IgG-positive subjects, 56.7% (95%CI: 50.0–63.2%) did not notice any of the 8 most common COVID-19 symptomsbetween March and end of June 2020, 18.0% (95%CI: 13.3–23.5%) reported 1 symptom, and 14.7% (95%CI: 10.5–19.9%) reported 2 symptoms. Similar responses were obtained in those with negative (*n* = 4196) and equivocal (*n* = 669) anti-SARS-CoV-2 IgG index (*p* = 0.954). The most common symptom was cough (27.4% of all respondents; 95% CI: 26.2–28.6%), but its rates did not differ significantly in relation to the IgG result (*p* = 0.731). Of the 8 symptoms, a significant correlation (*p* < 0.01) was found only for fever, which was reported by 17.1% (95%CI: 12.5–22.5%) of subjects with positive IgG index, 12.4% (95%CI: 11.4–13.4%) of those with a negative IgG index, and 9.0% (95%CI: 7.0–11.3%) of those with an equivocal IgG index.

No significant correlations were observed between the IgA+IgM result and the 8 analyzed COVID-19 symptoms between March and end of June 2020, with the difference close to statistical significance only for cough (*p* = 0.052).

## 4. Discussion

Our study is the first large cross-sectional SARS-CoV-2 screening survey performed among the personnel of the uniformed services in Europe. In our study population, the anti-SARS-CoV-2 IgM+IgA index was positive in nearly 9% of participants, and IgG index was positive in over 4% of participants, indicating a previous infection/exposure to SARS-CoV-2. Both indexes were positive in <1% of participants. Notably, all RT-PCR tests were negative, indicating no current SARS-CoV-2 infection, in all 5082 police employees in this study.

The relatively low individual overlap between positive results of the IgM+IgA and IgG indexes may be explained by the dynamics of various Ig class formation. During the course of SARS-CoV-2, IgM and/or IgA are detected first, followed by a longer-lasting IgG response. In most patients, seroconversion occurs between 7 and 14 days after the COVID-19 diagnosis [14]. However, the speed and strength of individual immune response may be variable, e.g., depending on the viral burden upon exposure, and in some people, detectable antibodies may be not generated after infection due to such factors as an underlying immune disorder, immunosuppression, or other reasons. It is more difficult to explain why we did not have any positive RT-PCR test results despite a 9% rate of positive IgM+IgA results, presumably indicating early infection. Due to the nature of police officers’ activities, one may speculate whether our results might reflect a contact, possibly recurrent, with low viral burden that would be insufficient to generate true infection/virus replication (as detectable by RT-PCR) but enough to trigger antibody (IgA/IgM) production. Studies show that while antibody responses may be undetectable or short-lived, memory T cell responses can persist for much longer and, indeed, SARS-CoV-2-specific T cells were detectable in antibody-seronegative-exposed family members and convalescent individuals with a history of asymptomatic or mild COVID-19 [15].

Obviously, it also possible that RT-PCR testing might have missed some active cases, and the antibody test used might have yielded some false positives. Many of our IgG-positive subjects reported some symptoms consistent with COVID-19, although only fever was significantly more common in those with positive IgG results compared to those without. There is a growing body of scientific data on nonrespiratorysymptoms of SARS-CoV-2 infection [16,17,18]. An analysis of COVID-19 symptom profiles showed that gastrointestinal symptoms (diarrhea/lack of appetite), neurological symptoms (loss of smell and/or taste), as well as chills, myalgia, headache, and fatigue, were commonly reported by patients with COVID-19 [16]. We can hypothesize that some IgG-positive subjects may have observed nonrespiratoryCOVID-19 symptoms and therefore did not report them to a physician or sanitary inspection.

Although the sensitivity of RT-PCR is very high, its overall diagnostic accuracy depends on the quality of sampling (nasopharyngeal swab) and subsequent sample handling. Antibody tests used might show cross-reactivity with other viruses, such as endemic coronavirus strains [19]. In a Dutch summary of various ELISA tests, the sensitivity of the Vircell IgG test at >14 days after illness onset was 97% in severe and 89% in mild/asymptomatic cases and the specificity was 94%. The sensitivity of the Vircell IgM+IgA test at >14 days after illness onset was 97% in severe and 70% in mild/asymptomatic cases and specificity was 82% [20]. In a recent study, the Vircell IgG test had a sensitivity of 98% and specificity of 83% [21]. In another unpublished study, the sensitivity was much lower (65% for IgG and 77% for IgM+IgA), whereasthe specificity was 96% for the Vircell IgG test and 83% for the Vircell IgM+IgA test [22].

Significant predictors of a positive IgM+IgA result included female gender, place of residence (town <20,000 inhabitants and city 20,000–500,000 inhabitants), and being a police officer (compared to civilian police employees).

Both positive and equivocal IgM+IgA results were significantly more common in women compared to men. Although the clinical course of COVID-19 is more severe in men [23] and the overall prevalence may be slightly higher in men, Italian data indicate that among women aged 20–49 years, the prevalence was higher in women, and only after age 50, women outnumbered by men [24]. This has been explained by the fact that younger women are more represented in jobs (health, education, hotels, and restaurants) exposing them to a higher risk of contagion due to personal contacts. Such an explanation seems less valid for police employees, but the higher rate in women may still be explained by gender differences in the nature of social contacts in general.

Regarding other predictors of positive IgM+IgA results, association with the local community size might be related to personal contacts with a larger number of people at risk of COVID-19, either related or unrelated to police employees’ professional activities, and being a police officer might involve a higher risk of exposure to COVID-19 compared to civilian employees. However, these might also be chance findings, as other variables potentially reflecting increased exposure, such as the estimated number of persons contacted daily and involvement in the surveillance of quarantined individuals and protection of public gatherings, did not emerge as significant predictors of positive IgM+IgA and IgG results.

The two significant predictors of positive IgG results were age ≥60 years and daily vaping. Use of e-cigarettes, both alone and in combination with conventional cigarette smoking, has been associated with increased virulence and inflammatory potential of respiratory pathogens in general [25] and with COVID-19 in particular [26]. However, the opposite results were also found for smoking. In a French cohort study, a lower proportion of participants with confirmed SARS-CoV-2 infection based on antibody detection was found in smokers compared to non-smokers [27].

Regarding the association with age, a number of studies provided somewhat divergent results compared to our findings. In a Dutch plasma donor study, antibodies against SARS-CoV-2 were detected significantly more often in younger people (18–30 years), which was thought to be related to different social behaviors and higher exposure to the virus before social distancing was implemented [28]. Similarly, the seroprevalence in those aged 20–49 years was significantly higher compared to those aged 50 years and older in Geneva [29]. In healthy blood donors in Milan, seroconversion to IgG was noted more commonly among younger subjects, while seroconversion to IgM was more common in older subjects [30].

In addition to positive IgG and IgM+IgA results, we also obtained equivocal results of these tests in a significant proportion of participants. In our study population, an equivocal IgM+IgA index was found in nearly 10% of participants, and IgG index was equivocal in 13% of participants. In general, repeated testing is recommended in case of an equivocal serological test result. In our study, testing was performed at a single time-point only, which constitutes a study limitation. An equivocal result indicates that antibodies were detected at a level close to the diagnostic threshold. Equivocal results may represent an early infection, detection of decreasing antibody level after a past infection, cross-reactivity with other viruses, an underlying immune disorder, immunosuppression, or other reasons. Our findings showed that 17.5% of participants had a positive or equivocal anti-SARS-CoV-2 IgG index. Based on this observation, we can hypothesize that due to the asymptomatic course of SARS-CoV-2 as well as due to the presence of nonrespiratory symptoms, the number of COVID-19 cases in the Polish population may be underestimated. Testing strategies for SARS-CoV-2 should be regularly revised to include new scientific data on nonrespiratory symptoms of COVID-19.

Regarding comparison of our findings to anti-SARS-CoV-2 seroprevalence estimates in the general population, no such data are available for Poland but they are emerging for other European countries. The estimated seroprevalence was about 5% in Spain (by point-of-care lateral-flow assay for anti-SARS-CoV-2 IgM/IgG and chemiluminescentmicroparticle immunoassay for IgG) [3] and up to 11% in Geneva (by another commercially available ELISA IgG test) [24], both countries with a several-fold higher per capita COVID-19 prevalence in the general population compared to Poland. No large population studies have been published with the VircellCOVID-19 ELISA IgG or IgM+IgA kits.

This study has several limitations. The overall response rate was slightly below 58% (based on laboratory test sampling), which might have introduced some bias in terms of potential exposure to SARS-CoV-2, e.g., employees with more duties and responsibilities might have been less likely to participate. To determine seroprevalence, we relied on a single type of a serological test and a single kit manufacturer, although previous comparisons of the VircellCOVID-19 ELISA IgG or IgM+IgA kits used do not indicate their inferior performance compared to other tests. The sensitivity and specificity of all currently available serological tests creates some potential for both false negatives and false positives, including cross-reactivity with other (corona) viruses, and false negatives are also possible with RT-PCR due to suboptimal nasopharyngeal swab technique or inadequate sample handling. The study protocol did not allow for retesting in individuals with an equivocal serological test result, which was dictated by feasibility issues and the intent to perform a single-time-point evaluation during a short period of up to 14–21 days.

## 5. Conclusions

The majority of Polish police employees are seronegative for SARS-CoV-2 infection. Most SARS-CoV-2 infections were asymptomatic or oligosymptomatic, and fever was the only symptom more often reported by IgG-positive subjects. E-cigarette use and older age (≥60 years) were associated with a higher risk of SARS-CoV-2 infection, which emphasizes the importance of quitting smoking to reduce the risk of infection. Relatively high proportions of study subjects were IgM+IgA-positive with negative RT-PCR, or had equivocal IgM+IgA or IgG indexes, an observation requiring further analyses.

## Figures and Tables

**Table 1:** Anti-SARS-CoV-2 IgM+IgA and IgG indexes in a group of 5082 police employees from Mazowieckie Province, Poland.

Variable		Anti-SARS-CoV-2 IgM+IgA index	Anti-SARS-CoV-2 IgG index
Mean		4.4	3.1
95%CI	LL	4.3	3.0
	UL	4.5	3.2
Median		3.5	2.6
Standard deviation (SD)		3.6	2.7
Minimum		0.0	0.0
Maximum		43.5	44.8
Range		43.5	44.8
Interquartile range		2.8	1.6
Percentiles	5	1.3	1.1
	10	1.6	1.4
	25	2.4	1.9
	50	3.5	2.6
	75	5.2	3.5
	90	7.6	4.6
	95	10.0	5.7

**Table 2:** Relationship between anti-SARS-CoV-2 IgG and anti-SARS-CoV-2 IgM+IgA indexes (values are added up in columns) (*n* = 5082).

Anti-SARS-CoV-2 IgA + IgM Index	Anti-SARS-CoV-2 IgG Index				
		Negative (<4) (*n* = 4196)	Equivocal (4–6) (*n* = 669)	Positive (>6) (*n* = 217)	Overall (*n* = 5082) *
		*n* (%)	*n* (%)	*n* (%)	*n* (%)
	Negative(<6)	3506 (83.6)	482 (72.0)	148 (68.2)	4136 (81.4)
	Equivocal (6–8)	371 (8.8)	95 (14.2)	30 (13.8)	496 (9.8)
	Positive (>8)	319 (7.6)	92 (13.8)	39 (18.0)	450 (8.9)

* percentages do not sum to 100% due to rounding.

**Table 3:** Effect of risk factors on positive results of anti-SARS-CoV-2 IgM+IgA (>8) and IgG indexes (>5)—a multivariate logistic regression model.

Variables	Anti-SARS-CoV-2 IgM+IgA Index				SARS-CoV-2 IgG Index			
	OR 95% CI				OR 95% CI			
	*p*	OR	Lower	Upper	*p*	OR	Lower	Upper
Gender (women)	0.000	1.742	1.377	2.203	0.572	1.104	0.784	1.554
Age group (30–44 years)	0.257	0.833	0.607	1.143	0.360	1.266	0.764	2.098
Age group (45–59 years)	0.578	0.905	0.637	1.286	0.550	1.184	0.680	2.061
Age group (60 and more)	0.243	0.617	0.275	1.386	0.005	3.309	1.442	7.595
Place of residence (town up to 20,000 residents)	0.012	1.526	1.099	2.119	0.934	0.980	0.611	1.572
Place of residence (city between 20,000 and 500,000 residents)	0.000	1.657	1.257	2.183	0.366	1.189	0.817	1.729
Place of residence (city above 500,000 residents)	0.111	1.276	0.946	1.721	0.461	1.159	0.783	1.716
Living with one or more persons (yes)	0.246	1.272	0.847	1.909	0.639	1.154	0.634	2.098
Presence of children aged 0–17 at home (daily)	0.581	0.936	0.739	1.185	0.541	1.111	0.793	1.557
Presence of children aged 0–17 at home (occasionally)	0.558	0.837	0.462	1.518	0.068	0.158	0.022	1.147
Police officer (yes)	0.047	1.411	1.004	1.981	0.317	1.279	0.790	2.068
Type of employment (office-based)	0.513	0.883	0.608	1.282	0.123	1.530	0.892	2.625
Type of employment (both office-based and fieldwork)	0.502	0.903	0.670	1.216	0.367	1.230	0.784	1.929
Average number of contacts per person perday (<5 persons)	0.123	2.119	0.815	5.510	0.791	1.311	0.177	9.679
Average number of contacts per person perday (5–9 persons)	0.572	0.801	0.371	1.729	0.540	1.593	0.359	7.072
Average number of contacts per person perday (10–19 persons)	0.879	1.057	0.516	2.167	0.305	2.115	0.506	8.846
Average number of contacts per person perday (20–49 persons)	0.903	1.046	0.513	2.132	0.228	2.404	0.578	9.995
Average number of contacts per person perday (50–100 persons)	0.522	0.778	0.362	1.675	0.178	2.722	0.634	11.678
Verification of compliance with mandatory quarantine rules (frequently)	0.786	1.045	0.761	1.435	0.976	0.993	0.633	1.559
Verification of compliance with mandatory quarantine rules (occasionally)	0.602	1.073	0.822	1.401	0.534	1.128	0.772	1.647
Protection of public gatherings > 50 persons (frequently)	0.261	0.780	0.505	1.204	0.815	1.070	0.605	1.893
Protection of public gatherings > 50 persons (1–2 times)	0.391	1.135	0.850	1.514	0.815	1.052	0.690	1.602
Presence of chronic condition (yes)	0.750	1.035	0.837	1.281	0.479	0.897	0.664	1.212
Tobacco smoking (daily)	0.572	1.077	0.833	1.393	0.182	0.772	0.528	1.129
Tobacco smoking (occasionally)	0.393	1.137	0.846	1.528	0.113	0.684	0.427	1.094
E-cigarette use/vaping (daily)	0.319	0.715	0.370	1.382	0.024	2.058	1.097	3.861
E- cigarette use/vaping (occasionally)	0.465	1.225	0.710	2.114	0.455	1.349	0.616	2.952
Heated tobacco use (daily)	0.858	1.056	0.581	1.919	0.549	0.730	0.260	2.045
Heated tobacco use (occasionally)	0.844	0.941	0.511	1.732	0.088	1.870	0.911	3.838
Foreign trip in the first half of 2020 (yes)	0.625	0.841	0.420	1.685	0.335	0.565	0.177	1.804
Number of COVID-19 cases in the poviat (administrative region) per 10,000 residents	0.102	1.033	0.994	1.073	0.202	1.035	0.982	1.090
Number of COVID-19 related deaths in the poviat (administrative region) per 10,000 residents	0.589	0.897	0.604	1.331	0.506	0.833	0.486	1.428
Constant	0.000	0.033			0.000	0.006		

Baseline = gender (male); age group (20–29 years); place of residence (rural); living alone; lack of children at home; civil employee; type of employment (fieldwork); average number of contacts per person perday (>100 person);verification of compliance with mandatory quarantine rules (no); protection of public gatherings > 50 persons (no); presence of chronic condition (no); tobacco smoking (no); e-cigarette use/vaping (no); heated tobacco use (no); foreign trip in the first half of 2020 (no).
